# Optimizing Experimental Design for Comparing Models of Brain Function

**DOI:** 10.1371/journal.pcbi.1002280

**Published:** 2011-11-17

**Authors:** Jean Daunizeau, Kerstin Preuschoff, Karl Friston, Klaas Stephan

**Affiliations:** 1 Wellcome Trust Centre for Neuroimaging, University College of London, London, United Kingdom; 2 Laboratory for Social and Neural Systems Research, Department of Economics, University of Zurich, Zurich, Switzerland; Indiana University, United States of America

## Abstract

This article presents the first attempt to formalize the optimization of experimental design with the aim of comparing models of brain function based on neuroimaging data. We demonstrate our approach in the context of Dynamic Causal Modelling (DCM), which relates experimental manipulations to observed network dynamics (via hidden neuronal states) and provides an inference framework for selecting among candidate models. Here, we show how to optimize the sensitivity of model selection by choosing among experimental designs according to their respective model selection accuracy. Using Bayesian decision theory, we (i) derive the *Laplace-Chernoff risk* for model selection, (ii) disclose its relationship with classical design optimality criteria and (iii) assess its sensitivity to basic modelling assumptions. We then evaluate the approach when identifying brain networks using DCM. Monte-Carlo simulations and empirical analyses of fMRI data from a simple bimanual motor task in humans serve to demonstrate the relationship between network identification and the optimal experimental design. For example, we show that deciding whether there is a feedback connection requires shorter epoch durations, relative to asking whether there is experimentally induced change in a connection that is known to be present. Finally, we discuss limitations and potential extensions of this work.

## Author Summary

During the past two decades, brain mapping research has undergone a paradigm switch. In addition to localizing brain regions that encode specific sensory, motor or cognitive processes, neuroimaging data is nowadays further exploited to ask questions about how information is transmitted through brain networks. The ambition here is to ask questions such as: “what is the nature of the information that region A passes on to region B”. This can be experimentally addressed by, e.g., showing that the influence that A exerts onto B depends upon specific sensory, motor or cognitive manipulations. This means one has to compare (in a statistical sense) candidate network models of the brain (with different modulations of effective connectivity, say), based on experimental data. The question we address here is how one should design the experiment in order to best discriminate such candidate models. We approach the problem from a statistical decision theoretical perspective, whereby the optimal design is the one that minimizes the model selection error rate. We demonstrate the approach using simulated and empirical data and show how it can be applied to any experimental question that can be framed as a model comparison problem.

## Introduction

The history of causal modeling of fMRI data in terms of effective connectivity began in the mid-1990's and has unfolded in two major phases (for reviews, see [1]–[2]). The first phase addressed the *optimization of connectivity estimates*. This involved optimising methods that exploited the information contained in fMRI time series and dealt with confounds such as inter-regional variability in hemodynamic responses. In this development, the community progressed from using methods originally developed for other types of data (such as structural equation modeling; [3]) to dynamic causal models, specifically tailored to fMRI [4]. The second phase concerned *optimization of model structure*, introducing Bayesian model selection methods to neuroimaging that are increasingly frequently used for selecting among competing models [5]. This paper goes beyond this and hopes to contribute to the initiation of a third phase. It describes a method for selecting experimental design parameters to minimize the model selection error rate, when comparing candidate models of fMRI data. This is the first attempt to formalize the *optimization of experimental design* for studying brain connectivity with functional neuroimaging data.

This paper describes a general framework for design optimization. Although we examine design optimization in the specific context of inferring effective connectivity and network structure from fMRI data, it should be noted that the approach is very general and not limited to any data acquisition technique, nor to any particular generative model. In brief, it can be used whenever one wishes to optimize experimental design for studying empirical responses by means of generative models.

To date, statistical approaches to experimental design for fMRI studies have focused on the problem of detecting regionally specific effects of experimental (e.g., cognitive, sensory or motor) manipulations [6]–[10]. This addresses the traditional question of *functional specialization* of individual areas for processing components of interest [11]. The associated statistical procedure involves testing for the significance of contrasts of effects of interest, encoded by regressors in the design matrix of a general linear model (GLM). The established approach to fMRI experimental design thus proceeds by extremising the experimental variance in summary statistics (e.g., GLM parameters estimates) at the subject level. This is typically done under (non statistical) constraints, such as psychological validity or experimental feasibility (see, e.g., [12]).

**Figure 1:**
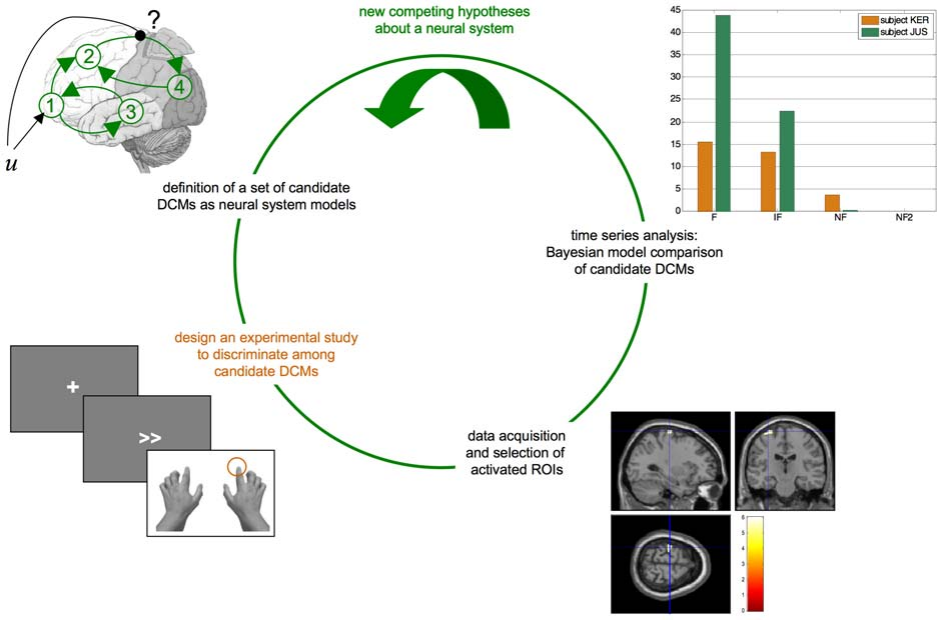
The DCM cycle. The DCM cycle summarizes the interaction between modelling, experimental work and statistical data analysis. One starts with new competing hypotheses about a neural system of interest. These are then embodied into a set of candidate DCMs that are to be compared with each other given empirical data. One then designs an experiment that is maximally discriminative with respect to the candidate DCMs. This is the critical step addressed in this article. Data acquisition and analysis then proceed, the conclusion of which serves to generate a new set of competing hypotheses, etc…

However, no attempt has been made so far to optimise experimental designs in relation to *functional integration*, i.e. the information transfer among activated brain regions. Here, the challenge is to identify context-dependent interactions among spatially segregated areas [13]. The key notion in this context is that optimizing the experimental design requires both a quantitative model that relates the experimental manipulation to observed network dynamics and a formal statistical framework for deciding, for example, whether or not a specific manipulation modulated some connection within the network (see Figure 1).

Dynamic Causal Modelling (DCM) was developed to exploit biophysical quantitative knowledge in order to assess the context-specific effects of an experimental manipulation on brain dynamics and connectivity [4]. Typically, DCM relies upon Bayesian model comparison to identify the most likely network structure subtending observed fMRI time series within regions of interest. We refer the interested reader to [14] for a critical review on the biophysical and statistical foundations of the DCM framework. At present, DCM is the most suitable framework within which to address the problem of optimizing the experimental design to infer on brain network structure. This is because it is based upon a generative model that describes how experimental manipulations induce changes in hidden neuronal states that cause the observed measurements. This is in contrast to other network models based on functional connectivity that simply characterise the surface structure or statistical dependencies among observed responses [15].

In this paper, we argue that one should choose among experimental designs according to their induced model selection error rate and demonstrate that this can be done by deriving an information theoretic measure of discriminability between models. We first derive and evaluate the *Laplace-Chernoff risk*, both in terms of how it relates to known optimality measures and in terms of its sensitivity to basic modelling choices. The ensuing framework is very general and can be used for any experimental application that rests upon Bayesian model comparison. We then use both numerical simulations and empirical fMRI data to assess standard design parameters (e.g., epoch duration or site of transcranial magnetic stimulation). In brief, we formalize the intuitive notion that the best design depends on the specific question of interest. *En passant*, we also identify the data features that inform inference about network structure. Finally, we discuss the limitations and potential extensions of the method.

## Methods

Bayesian model selection is a powerful method for determining the most likely among a set of competing hypotheses about (models of) the mechanisms that generated observed data. It has recently found widespread application in neuroimaging, particularly in the context of dynamic causal modelling (DCM). However, so far, optimizing experimental design has relied upon classical (frequentist) results that apply to parameter estimation in the context of the general linear model. This section presents the derivation of the *Laplace-Chernoff risk*, which serves as a proxy to the model selection error rate. The emphasis here is on model selection, rather than parameter estimation. This is important, because the former problem cannot, in general, be reduced to the latter, for which most formal optimality criteria have been designed [16]. We thus outline the theory, which involves: (i) deriving a Bayesian decision theoretic design optimality score: this can be understood, in information theoretic terms, as expected model discriminability; (ii) disclosing its relationship to classical (frequentist) design optimality and (iii) inspecting its sensitivity to basic modelling assumptions.

### Bayesian model comparison

To interpret any observed data 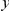 with a view to making predictions based upon it, we need to select the best model 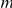 that provides formal constraints on the way those data were generated; (and will be generated in the future). This selection can be based on (Bayesian) probability theory to identify the best model in the light of data. This necessarily involves evaluating the model evidence or marginal likelihood 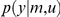:
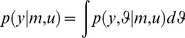
(1)where 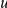 is the (known) experimental manipulation (or design) and the generative model 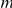 is defined in terms of a likelihood 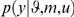 and prior 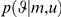 on the unknown model parameters, 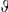, whose product yields the joint density by Bayes rule:
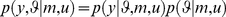
(2)Generally speaking, 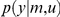 is a density over the set of all possible datasets 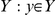 that can be generated under model 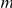 and experimental design 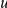. Having measured data 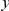, Bayesian model comparison relies on evaluating the posterior probabilities 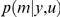 of models 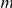 belonging to a predefined set 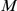:
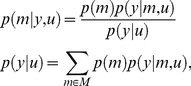
(3)The reason why 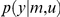 is a good proxy for the plausibility of any model 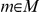 is that the data 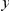 sampled by the experiment are likely to lie within a subset of 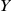 that is highly plausible under the model whose predictions are the most similar to the true generative process. However, there is a possibility that the particular experimental sample 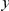 could end up being more probable under a less reasonable model. This ‘model selection error’ could simply be due to chance, since 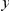 is sampled from a (hidden) probability distribution. In what follows, we focus on inferential procedures based on Bayesian model selection (e.g., DCM studies, see below). The experimental design should then minimize the expected model selection error. We now turn to a formal Bayesian decision theoretical approach for design optimization (we refer the interested reader to [17] for an exhaustive review).

### The Chernoff bound to the model selection error rate

Following [18], we consider the following decision theoretic problem. A design 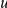 must be chosen from some set 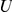 and data 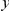 from a sample space 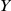 is observed. Based on 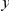, a model 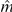 will be chosen from the comparison set or model space 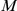. Note that the decision is in two parts: first the selection of the design 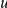, and then the model selection 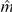. Before the experiment is actually performed, the unknown variables are the models 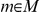 and the data 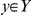. Within a Bayesian decision theoretic framework (see e.g., [19]), the goal of the experiment is quantified by a loss function 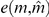, which measures the cost incurred in making decision 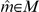 (the selected model) when the hidden model is 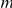. Note that obviously, no model is ‘true’ (or ‘false’): it is an imperfect approximation to reality, whose imperfections can, in certain circumstances, become salient; by ‘hidden model’, we mean ‘the model that is the least imperfect’. Following the Neyman-Pearson argument for hypothesis testing [20], we define the model selection error or loss 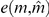 as follows:
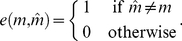
(4)According to Bayesian decision theory, the optimal decision 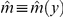 is the one that minimizes the so-called *posterior risk*, i.e. the expected model selection error, given the observed data 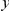:
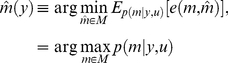
(5)where the expectation is taken over the model posterior distribution 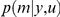. The optimal decision rule 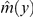 depends on the observed data 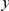, whose marginal density 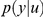 depends on the experimental design 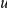. A model selection error might still arise, even when applying the optimal model selection in equation 5. Note that the probability 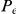 of selecting an erroneous model, given the data and having applied the optimal model selection rule is simply given by:
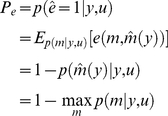
(6)where we have used 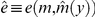, for the potential error we make when selecting the optimal model 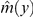. Equation 6 means that the probability of making a model selection error is determined by the experimental evidence in favour of the selected model. Thus, repetitions of the same experiment might not lead to the same model being selected because of the variability of the posterior probability distribution over models 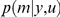, induced by the sampling process.

In this context, the task of design optimization is to reduce the effect of the data sampling process upon the overall probability of selecting the wrong model. This means we have to marginalize the probability 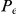 of making an error 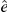 over the data sample space 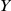. Note that design optimization is the only Bayesian problem where it is meaningful to average over the sample space 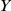. This is because the experimental sample 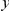 has not yet been observed, which makes the decision theoretic principle of averaging over what is unknown valid for 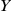. More formally, the potential error 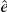 is the loss in our design decision theoretical problem, and the model selection error rate 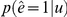 is the *design risk* for Bayesian model selection. We define the optimal design (for Bayesian model selection) as the design 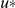 that minimizes the design risk; i.e. the expectation of 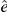 under the marginal prior predictive density 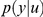:
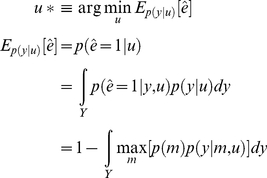
(7)where we have used the expression for the error probability 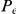 in equation 6. The integrand in equation 7 switches from one model to another one as one spans the data sample space 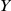. Unfortunately, this means that the error rate 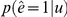 has no analytical close form, and might therefore be difficult to evaluate. Instead, we propose to minimize an information theoretic criterion 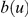 that yields both upper and lower bounds to the above error rate [21]:
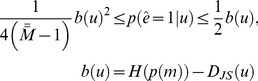
(8)where 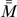 is the cardinality of the model comparison set 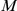, 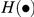 is the Shannon entropy and 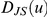 is the so-called *Jensen-Shannon divergence* (see, e.g., [22]), which is an entropic measure of dissimilarity between probability density functions:
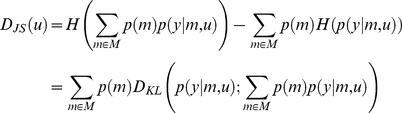
(9)where 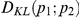 is the Kullback-Leibler divergence between the densities 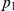 and 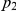. Note that the Jensen-Shannon divergence is symmetric, nonnegative, bounded by 1 (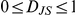) and equal to zero if and only if all densities are equal. It is also the square of a metric (that of convergence in total variation).

In the context of classification or clustering, 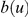 is known as the *Chernoff bound* to the classification error rate [21]. Note that, since the prior distribution 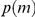 over model space 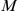 is independent of design 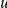, minimizing 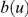 with respect to 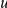 corresponds to maximizing 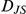 with respect to 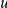. From equation 9, one can see that 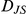 is the difference between the entropy of the average prior predictive density over models minus the average entropy. In this setting, entropy can be thought of as average self information over models. Maximising 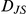 minimises the dependencies among the prior predictive densities. Informally, one could think of this as orthogonalising the design, in the same way that one would orthogonalise a covariance matrix, namely minimise the covariances (the first term in equation 9 – first line) under the constraint that the variances are fixed (second term in equation 9 – first line). The second line in equation 9 gives yet another interpretation to the Jensen-Shannon divergence: it is the average Kullback-Leibler divergence between each prior predictive density and the average prior predictive density. It is a global measure of dissimilarity of the prior predictive densities; maximizing 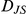 thus separates each model prediction from the others. In turn, this means that the optimal design 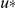 is the one that is the most discriminative, with respect to the prior predictive density of models included in the comparison set.

In summary, we have derived the Bayesian decision theoretic design optimization rule that minimizes the model selection error rate. We have then proposed an information theoretic bound, which relies upon maximizing the discriminability of model predictions with respect to experimental design. We now turn to a specific class of generative models, that of nonlinear Gaussian likelihood functions, which is a class of generative models that encompasses most models used in neuroimaging data analyses.

### Nonlinear Gaussian models and the approximate Laplace-Chernoff risk

In the following, we will focus on the class of nonlinear Gaussian generative models. Without loss of generality (under appropriate nonlinear transformations), this class of models has the following form:
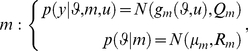
(10)where 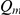 is the covariance matrix of the residual error 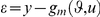, 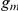 is the (deterministic) observation mapping of model 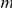 and 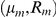 are the prior mean and covariance of the unknown parameters 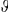 (under model 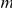).

For this class of models, and using an appropriate Taylor expansion of the observation mapping, one can derive (see Text S1) an analytical approximation to the lower Chernoff bound to the model selection error rate 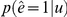:
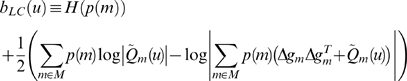
(11)where 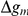 and 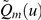 are defined as follows:
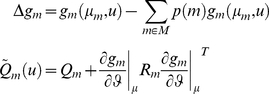
(12)In the following, we will refer to 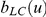 as the *Laplace-Chernoff risk*. In the following, we will show that, under mild conditions, the Laplace-Chernoff risk is monotonically related to the model selection error rate 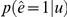, and is therefore a valid proxy.

So far, we have considered the problem of selecting a single model from a set of alternatives. However, we may want to compare families of models, irrespective of detailed aspects of model structure [23]. This optimization of experimental design for comparing *model families* is described in Text S3.

### Relationship to classical design efficiency

**Figure 2:**
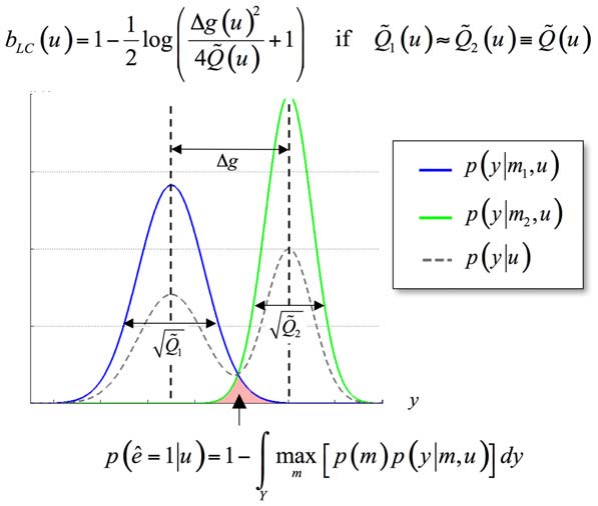
Selection error rate and the Laplace-Chernoff risk. The (univariate) prior predictive density of two generative models 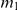 (blue) and 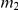 (green) are plotted as a function of data 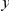, given an arbitrary design 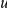. The dashed grey line shows the marginal predictive density 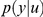 that captures the probabilistic prediction of the whole comparison set 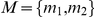. The area under the curve (red) measures the model selection error rate 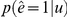, which depends upon the discriminability between the two prior predictive density 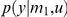 and 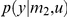. This is precisely what the Laplace-Chernoff risk 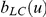 is a measure of.

The Laplace-Chernoff risk is simple to compute and interpret. For example, with 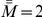 models and assuming that (i) both models are a priori equally likely, and (ii) both prior predictive densities have similar variances, i.e.: 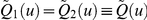, the Laplace-Chernoff risk is given by:
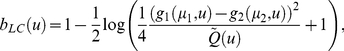
(13)Equation 13 shows that the Laplace-Chernoff bound 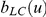 is a simple contrast resolution measure, in a signal detection theory sense (see Figure 2). Another perspective would be to think of it as a (log-transformed) t-test of the mean difference under two designs. From equation 13, one can see that the Laplace-Chernoff bound tends to one (i.e. the upper bound on the error rate 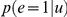 tends to 0.5) whenever either the difference 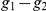 between the first-order moments of the prior predictive densities goes to zero or their second-order moment 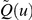 goes to infinity.

Optimizing the design 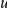 with respect to 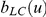 thus reduces to discriminating the prior predictive densities, either by increasing the distance between their first-order moments, and/or by decreasing their second-order moments. Although this is not directly apparent from the general mathematical form of the Laplace-Chernoff bound (c.f. Equation 11), this intuition generalizes well to an arbitrary number of models and data dimensions.

To demonstrate the properties of the Laplace-Chernoff bound, we will compare it with the classical design efficiency measure, under the general linear model (GLM), which is a special case of equation 10:
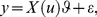
(14)where 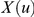 is the design matrix. The classical efficiency of a given contrast of parameters 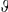 is simply a function of the expected variance of the estimator of 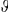. For example, when a contrast is used to test the null assumption 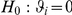, the classical efficiency 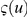 is [10]:
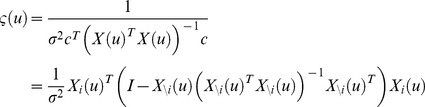
(15)where the contrast vector 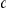 has zero entries everywhere except on its 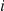 ^th^ element, 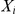 is the 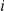 ^th^ column of the design matrix 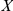, 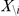 is 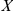 without 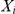 and 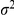 is the noise variance. Since decreasing the variance of the parameter estimates increases the significance for a given effect size, optimizing the classical efficiency 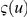 simply improves statistical power; i.e., the chance of correctly rejecting the null. Although there are other design efficiency metrics (see, e.g., [4]), this design efficiency measure, so-called C-optimality, is the one that is established in the context of standard fMRI studies [10].

The equivalent Bayesian test relies on comparing two models, one with the full design matrix 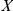 and one with the reduced design matrix 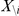. Under i.i.d. Gaussian priors for the unknown parameters 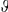 and flat priors on models 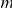, one can show (see Text S2) that the Laplace-Chernoff risk 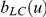 simplifies to the following expression:
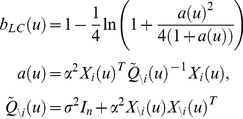
(16)where 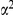 is the prior variance of the unknown parameters. Text S2 demonstrates that the optimal design at the frequentist limit (non-informative priors, i.e.: 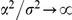) is the design that maximizes the classical design efficiency measure:
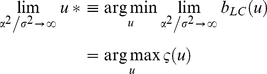
(17)In brief, under flat priors, optimizing the classical efficiency of the design minimizes the model selection error rate for the equivalent Bayesian model comparison. This is important, since it allows one to generalise established experimental design rules to a Bayesian analysis under the GLM.

This result generalizes to any classical null hypothesis testing, which can be cast as a comparison of nested models (as above), under appropriate rotations of the design matrix. However, there are model comparisons that cannot be performed within a classical framework, such as non-nested models. This means that even at the frequentist limit and for linear models, equation 16 is more general than equation 15.

Note that this equivalence is only valid at the limit of uninformative priors. For linear generative models, such as the GLM, this may not be a crucial condition. However, priors can be crucial when it comes to comparing nonlinear models. This is because *a priori* implausible regions of parameter space will have a negligible influence on the prior predictive density, even though their (conditional) likelihood may be comparatively quite high (e.g., a multimodal likelihood).

### Tightness of the Laplace-Chernoff bounds

**Figure 3:**
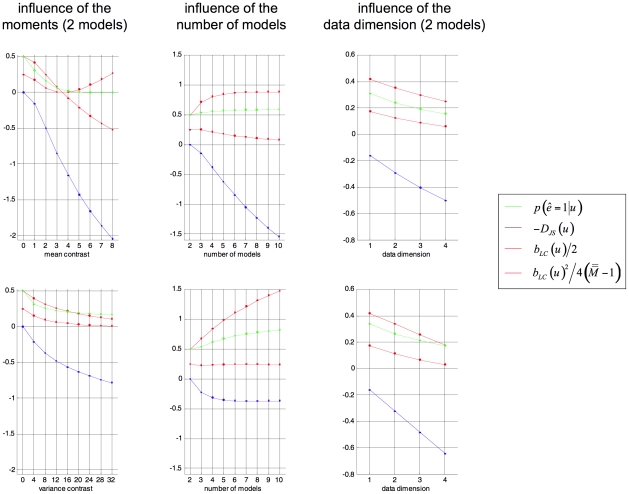
Tightness of the Laplace-Chernoff bounds. The figure depicts the influence of a moment contrast between two prior predictive densities (left column), the number of models (middle column) and the data dimension (right column) onto the exact error rate 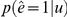 (green) and the Laplace-Chernoff risk 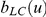 (upper bound: solid red, lower bound: dashed red). This is assessed in terms of a mean shift (left inset) and a variance scaling (right inset). The blue lines depict the approximate Jensen-Shannon density 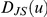 (see equations 8, 9 and 11 in the main text and equation A1.5 in Text S1).

We now examine the tightness of the Laplace-Chernoff bounds on the selection error rate. More precisely, we look at the influence of the moments 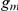 and 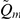 of the prior predictive densities 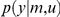, the dimension of the data (i.e. the sample size 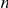) and the number of models 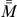 in the comparison set (see Figure 3).

We will first focus on the comparison of two models 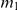 and 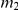, whose respective prior predictive densities were assumed to be univariate Gaussian (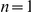), with mean 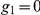 and variance 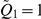 for 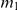 and varying moments for 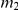 (see below). For this low-dimensional case, solving Equation 7 with numerical integration is possible and yields the exact selection error rate 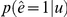 for each model comparison. The left column in Figure 3 depicts the Laplace-Chernoff bounds as a function of the first order moment 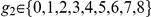 (bottom inset) and as a function of the second order moment 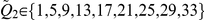 (upper inset) of 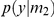, when comparing 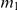 versus 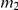. One can see that the error rate 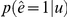 decreases as the moment contrast (either a mean shift or a variance scaling) increases. In addition, the Laplace-Chernoff risk 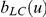 is related monotonically to the error rate 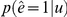. However, there is a moment contrast above which the upper bound breaks down, in the sense that the condition 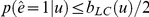 is not satisfied.

Second, we varied the number of models 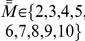 in the comparison set, where each model was characterized by a univariate Gaussian prior predictive density (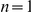). The middle column in Figure 3 depicts the Laplace-Chernoff bounds as a function of 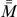, where 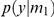 had mean 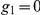 and variance 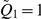, and any new model 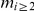 had a mean shift of 1 (bottom inset) or a variance scaling of 4 (upper inset), with respect to the preceding one. This ensured that the discriminability between two neighbouring models was comparable. One can see that the error rate 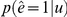 increases as the number of models 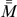 increases and that the Laplace-Chernoff risk 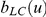 follows monotonically. However, there may be a number of models above which the upper bound becomes vacuous, in the sense that the condition 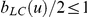 is not satisfied (although the bounding condition seems to be preserved).

Finally, we varied the sample size 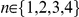, when comparing models 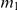 and 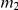. The right column in Figure 3 depicts the Laplace-Chernoff bounds as a function of 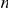, where 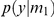 had mean 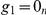 and variance 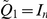 and model 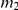 had a mean shift of 1 in each dimension; i.e., 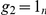
−(bottom inset) or a variance scaling of 4 – i.e. 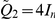
−(upper inset). This ensured that the discriminability increased monotonically with the sample size. One can see that the error rate 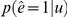 decreases as the sample size 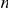 increases and that the Laplace-Chernoff risk 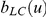 again changes monotonically. However, again, there is a sample size above which the upper bound breaks down; in the sense that the condition 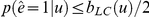 is not satisfied. This situation is very similar to increasing the mean or variance contrast; i.e., increasing the sample size can be thought of as increasing the discriminability of models in the comparison set.

Taken together, these results suggest that the Laplace-Chernoff risk 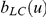 is a good proxy for the model selection error rate; in that there is a monotonic mapping between the two quantities. Furthermore, the upper bound becomes tightest for the worst (least decisive) model comparisons. This is important, because this means that the approximation by the Laplace-Chernoff risk is best when we most need it most. However, the Laplace-Chernoff risk can become more liberal than the true error probability. The subtle point here is that the model number and their discriminability have an opposite effect on the tightness of the bound. We will further examine the quality of the Laplace-Chernoff bounds in the context of effective connectivity analysis with DCM in the next section.

## Results

### Design risk for DCM: preliminary considerations

In Dynamic Causal Modelling (DCM), hemodynamic (fMRI) signals arise from a network of functionally segregated sources; i.e., brain regions or neuronal sources. More precisely, DCMs rely on two processes:

- DCMs describe how experimental manipulations (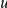) influence the dynamics of hidden (neuronal and hemodynamic) states of the system (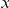). This is typically written in terms of the following ordinary differential equation (the *evolution equation*):
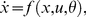
(18)where 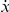 is the rate of change of the system's states 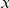, 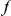 summarizes the biophysical mechanisms underlying the system's temporal evolution and 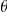 is a set of unknown evolution parameters. In particular, the system states include ‘neural’ states, which are driven by the experimental stimuli and cause variations in the fMRI signal. Their evolution function is given by [4], [24]:
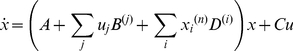
(19)The parameters of this neural evolution function include a between-region coupling (matrix 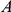), input-dependent coupling modulation (matrices 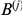), input driving gains (matrix 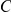) and gating effects (matrices 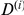).
- DCMs map the system's hidden states (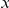) to experimental measures (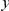). This is typically written as the following static *observation equation*:
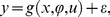
(20)where 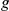 is the instantaneous non-linear mapping from system's states to observations, 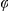 is a set of unknown observation parameters and 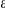 are model residuals.

Note that the ensuing dynamic causal model includes the effect of the hemodynamic response function that can change over brain regions. Equations 18 and 20 can be compiled into a nonlinear Gaussian generative model (similar in form to equation 10), which, given experimental data 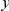, can then be inverted using a variational Bayesian approach. This scheme provides an approximate posterior density 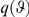 over the unknown model parameters 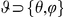 and a lower bound 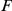 (free energy) to the models log-evidence or marginal likelihood 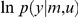. The free energy is used for comparing DCMs that represent competing hypotheses about network mechanisms, specified in terms of network structure and the modulation of specific connections. See [14] for a critical review of the biophysical and statistical foundations of DCM.

In brief, DCMs belong to the class of generative models for which we have derived the Laplace-Chernoff design risk (Equation 11). In what follows, we will evaluate the proposed method in the context of network discovery with DCM. First, we will evaluate the quality of the Laplace-Chernoff bound. Having established the conditions for this bound to hold, we will then focus on optimal designs for some canonical questions. These two steps will be performed using Monte-Carlo simulations. Finally, we will turn to an empirical validation of the simulation results, using data acquired from two subjects performing a simple finger-tapping experiment in the fMRI scanner.

### Evaluation of the model selection error bounds

**Figure 4:**
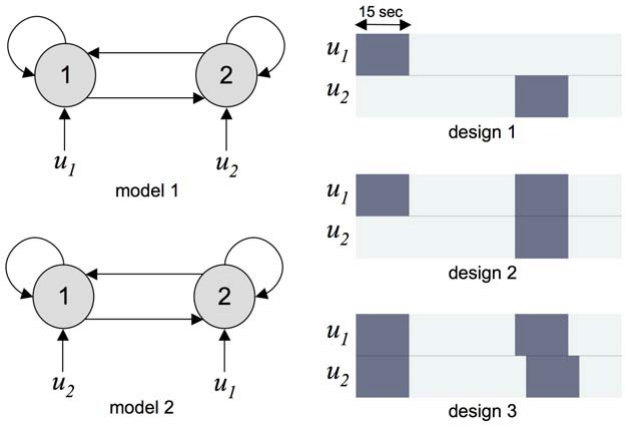
Evaluation of the Laplace-Chernoff bounds: DCM comparison set and candidate designs. This figure summarizes the Monte-Carlo simulation environment of section “Evaluation of the model selection error bounds” we used for evaluating the Laplace-Chernoff bounds in the context of network identification. The comparison set is shown on the left. It consists of two models that differ in terms of where the two inputs 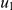 and 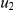 enter the network. The three candidate designs are shown on the right. They consist of three different stimulation sequences, with different degrees of temporal correlation between the two inputs.

In this section, we ask whether the Laplace-Chernoff bounds on the error rate 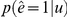 are consistent. This can be addressed by comparing the predicted bounds to the observed model selection error rate across repetitions of the same experiment. We have conducted a series of Monte-Carlo simulations, which reproduced the main characteristics of the finger-tapping task used in the section on empirical validation. Specifically, we considered two candidate DCMs (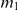 and 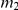) that consist of two (reciprocally connected) regions, each driven by a different experimentally controlled input (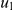 and 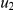, respectively). The two models differed in which of the two inputs drove which region. We then examined Bayesian model comparison (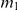 versus 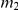) under three designs 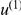, 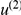 and 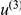, which differed in the temporal dynamics of the two inputs they affect. More precisely, we increase the correlations between the two stimuli: 
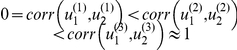
. This makes it increasingly difficult to disambiguate the respective impact of each input on network dynamics. In turn, we expect these three designs to be increasingly risky when discriminating among the two candidate DCMs. Figure 4 summarizes the structure of the two DCMs and shows the time course of the three designs' stimulation paradigms (experimental inputs).

To explore a range of plausible scenarios, we varied the following four factors to simulate 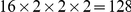 datasets 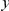:

- Sixteen random realisations of the residuals 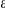, which were sampled according to their prior density 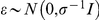, where 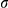 is the residuals' precision (see below).
- Two levels of effective connectivity 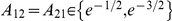. This factor was used to manipulate the discriminability of the two models. This is because it is more difficult to determine the respective contribution of the two inputs to the responses in each region as the effective connectivity increases.
- Two generative models (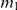 and 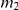). This factor is required because the selection error probability is symmetric with respect to the model that generated the data.
- Two levels of noise, i.e.: 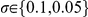, which correspond to realistic signal-to-noise ratios. This factor controls the overall discriminability of the two models, by scaling non-specific processes contributing to the data. Note that the approximate error probability bounds are conditional on the expected noise precision.

**Figure 5:**
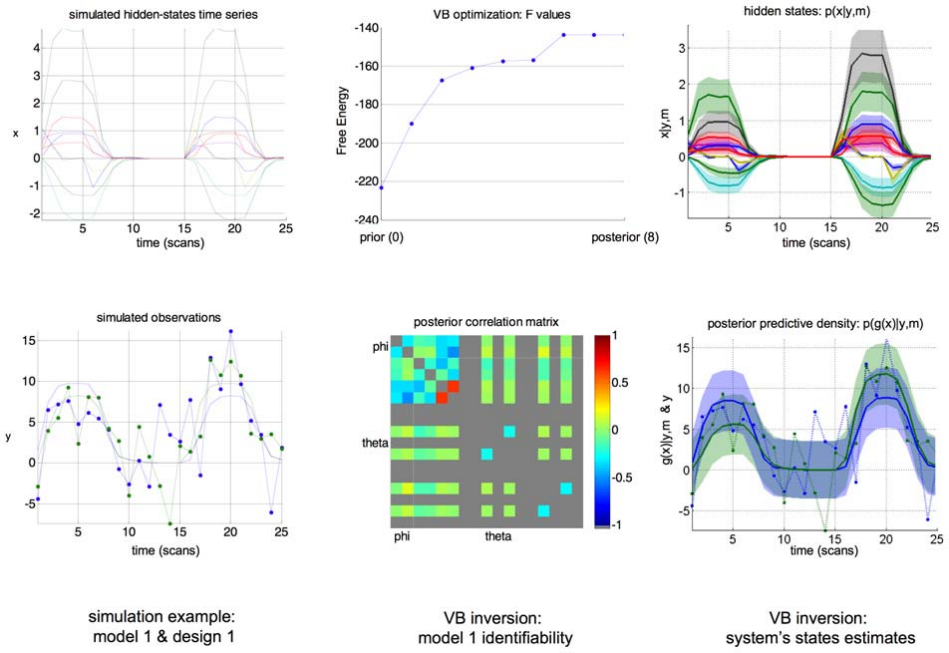
Evaluation of the Laplace-Chernoff bounds: simulated data and VB inversion. Upper-left: simulated (neural and hemodynamic) states dynamics 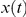 as a function of time under model 1 and design 1 (two regions, five states per region). Lower-left: simulated fMRI data (blue: region 1, green: region 2). Solid lines show the observable BOLD changes 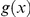 (without noise) and dashed lines show the actual noisy time series 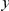 that are sent to the VB inversion scheme. Upper-middle: the iterative increase in the lower bound to the model evidence 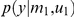 (free energy) as the VB inversion scheme proceeds (from the prior to the final posterior approximation), under model 1. Lower-middle: Posterior correlation matrix between the model parameters. Red or blue entries indicate a potential non-identifiability issue and grey entries are associated with fixed model parameters. Upper-right: approximate posterior density over (neural and hemodynamic) states 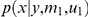. The first two moments of the density are shown (solid line: mean, shaded area: standard deviation). Lower-right: approximate posterior predictive density 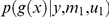 and data time series.

Each dataset was inverted (fitted) under both models (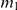 and 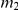), using a variational Laplace scheme [25], and Bayesian model selection was performed using the free energy approximation to the log evidence. We used shrinkage i.i.d. Gaussian priors for evolution and observation parameters (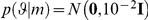), and weakly informative Gamma priors for the precision (scale parameter equal to the simulated noise precision and unit shape parameter). The same priors were used to derive the Laplace-Chernoff bounds. Figure 5 depicts a typical simulation and model inversion.

We counted the number of times the selected model 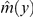 was different from the simulated ground truth. Averaging over the first three factors, this yielded a Monte-Carlo estimate 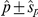 of the selection error rate 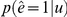, where 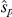 is the standard deviation of the Monte-Carlo estimate, for each of the three designs 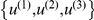 and each of the two noise levels 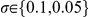.

**Figure 6:**
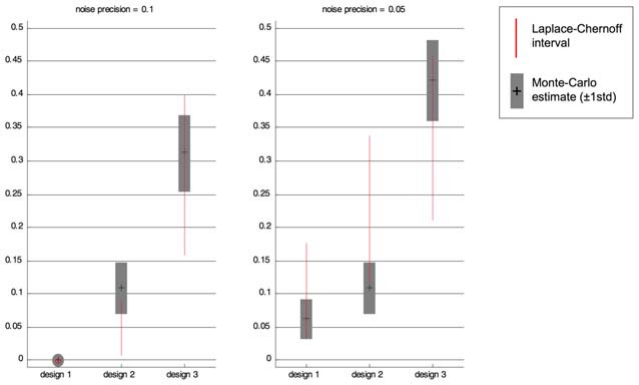
Evaluation of the Laplace-Chernoff bounds: Monte-Carlo results.

Figure 6 presents a graphical comparison between the Monte-Carlo confidence interval 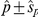 on the error rate with the Laplace-Chernoff bounds. First, one can see that the average selection error probability (both predicted and estimated) decreases with the residual precision 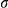. This is expected: as signal-to-noise ratio increases, the more discriminative evidence favouring one model or another exists in the data. Second, one can see that both estimated and predicted intervals on the selection error probability agree quantitatively: more precisely, the Monte-Carlo confidence intervals 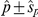 always intersect with the Laplace-Chernoff bounds; and for both residual precision levels, both the Monte-Carlo estimate of the error rate and the Laplace-Chernoff risk equally rank the three designs: 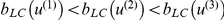 and 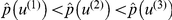. This means that for these levels of noise and sample sizes, the Laplace-Chernoff bound is in good agreement with the design risk. However, this quantitative agreement might break down for higher sample sizes or noise precision (cf. section “Tightness of the Laplace-Chernoff bounds” and Figure 3).

### Laplace-Chernoff risk for canonical network identification questions

**Figure 7:**
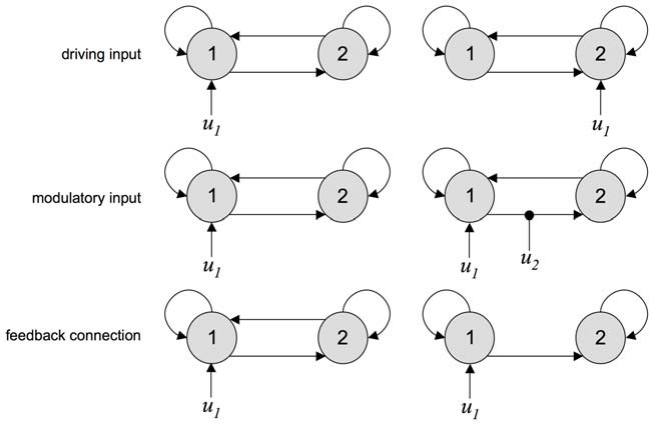
Canonical network identification questions: DCM comparison sets. This figure depicts the three canonical DCM comparison sets, each of which consists of two variants of a simple two-region network. Upper-row: driving input; middle-row: modulatory input; Lower-row: feedback connection.

The aim of this section is twofold: to investigate the sensitivity of the Laplace-Chernoff risk to the prior densities, and to demonstrate the importance of the model comparison set. We thus chose three “canonical network identification questions”, i.e. three simple model comparison sets that represent typical questions addressed by DCM. Figure 7 shows these model sets, each of which is composed of two variants of a two-region network:

- *Driving input*: the two DCMs differ in terms of where the input 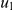 enters the network.
- *Modulatory input*: the two DCMs differ in terms of whether or not the experimental manipulation 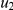 modulates the feedforward connection from node 1 to node 2.
- *Feedback connection*: the two DCMs differ in terms of whether or not there is a feedback connection from node 2 to node 1.

**Figure 8:**
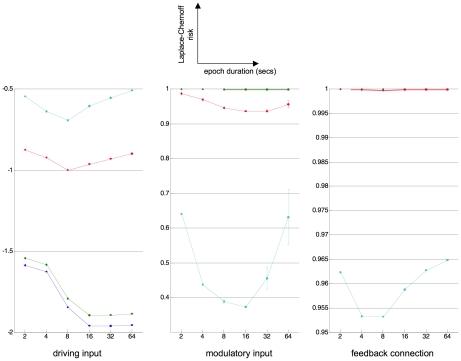
Canonical network identification questions: optimal epoch duration. This figure shows plots of the average (across jitters) Laplace-Chernoff risk as a function of epoch duration (in seconds) and prior expectation 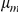 of neural evolution parameters, for the three canonical comparison sets (left: driving input, middle: modulatory input, right: feedback connection). Blue: 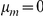, green: 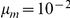, red: 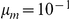 and magenta: 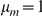. Error bars depict the variability (one standard deviation) induced by varying jitters in the stimulation sequence.

We then compared different experimental designs, considering blocked on/off (square wave) designs, and varying the epoch duration within the range 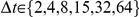. Comparing the Laplace-Chernoff risk of such designs allows one to identify the optimal epoch duration for each network identification question. In addition, we varied the first-order moment of the prior densities over neural evolution parameters 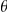 within the range 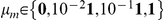, where 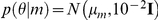. As above, we used i.i.d. shrinkage priors for the hemodynamic evolution and observation parameters (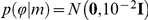) and non-informative Gamma priors for the noise precision (with scale parameter equal to 10^−1^ and unit shape parameter). This allowed us to evaluate the influence of the expected coupling strength on design optimisation. The average time interval between two blocks was held at 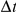, but a random jitter was added to this average inter-block time interval. For each 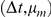 pair, we randomly drew sixteen stimulation sequences 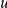. Figure 8 depicts the average (across random jitters) Laplace-Chernoff risk as a function of both epoch duration and prior mean of the evolution parameters, for the three canonical network identification questions.

First, one can see that the main effect of the prior mean is to increase the discriminability among the models in the comparison set, except in the ‘driving input’ case. This means that, in general, the discriminative power of the design increases with the expected effect size. This does not work for the ‘driving input’ case, however, because of the feedback connections, which tend to synchronize the two regions of the network and thus blur the distinction between the predictions of the two models.

**Figure 9:**
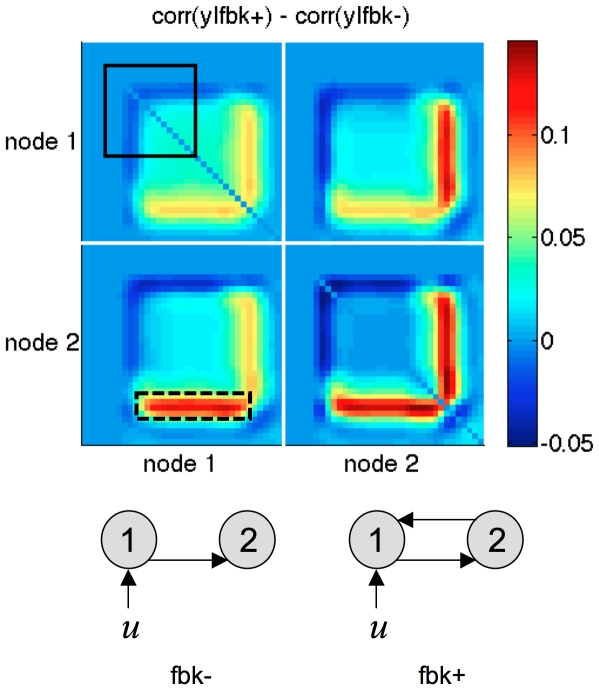
The signature of feedback connections. The figure depicts the difference in the data correlation matrices induced by two network structures (model fbk-: without feedback, model fbk+: with feedback). Red (respectively, blue) entries indicate an increase (respectively, a decrease) in the correlation induced by adding a feedback connection from node 2 to node 1. Each block within the matrix corresponds to a node-to-node temporal correlation structure (upper-left: node 1 to node 1, lower-right: node 2 to node 2, upper-right/lower-left: node 1 to node 2). For example, the dashed back box reads as follows: adding the feedback connection increases between activity in node 2 at the end of the block and node 1 during the whole block. The solid black box indicates the time interval, during which input 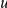 to node 1 was ‘on’. Note that its effect onto the two-region network dynamics is delayed, due to the hemodynamic response function.

Second, the optimal epoch duration depends on the question of interest. For example, the optimal epoch duration is 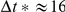 seconds, when asking whether there is a modulatory input or where the driving input enters the network, which is close to the optimal epoch duration for classical (SPM) activation studies [19]. Strictly speaking, note that in the “driving input” case, the optimal epoch duration additionally depends upon the expected coupling strength: about 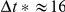 seconds for low coupling and 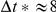 seconds for high coupling. On average however, the optimal epoch duration is much shorter when trying to disclose the feedback connection (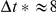 seconds). This might be due to the fact that a feedback connection mostly expresses itself during the transient dynamics of the network's response to stimulation (moving from or returning to steady-state). Decreasing the epoch duration increases the number of repetitions of such transitions, thus increasing the discriminative power of the design. To test this, we looked at the difference between the covariance matrices of the prior predictive densities of a model with and without feedback, respectively. This difference is depicted on Figure 9, for the highest prior mean of evolution parameters: i.e., highest coupling strength.

One can see that a feedback connection expresses itself when the system goes back to steady-state and increases the correlations between the nodes. This specific contribution to the statistical structure of the fMRI data is what DCM uses to infer the presence of a feedback connection.

Finally, one can see that there is a clear difference in the average Laplace-Chernoff risk between the three canonical network identification questions. This speaks to the overall discriminability of the models, within each comparison set. For example, it is easier to decide where the driving input enters the network (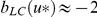) than to detect a modulatory effect (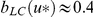) or a feedback connection (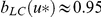). However, when optimizing other design parameters unrelated to epoch duration (e.g., sampling rate), this ranking could change.

### Investigating psycho-physiological interactions with DCM

In the context of DCM for fMRI, there are many design parameters one may want to control. These include, but are not limited to: (i) the physics of MRI acquisition (e.g., sampling rate versus signal-to-noise ratio), (ii) sample size, (iii) stimulus design and timing (e.g., categorical versus parametric, epoch duration, inter-stimulus time interval), and (iv) the use of biophysical interventions (e.g., transcranial magnetic stimulation, TMS). Assessing all these design parameters is well beyond the scope of the present article, and will be the focus of forthcoming publications. In this section, we demonstrate the use of the Laplace-Chernoff risk in the context of (iii) or (iv). This is addressed by two simulations that recapitulate common experimental questions of interest: characterizing psycho-physiological interactions (PPI) and using TMS for network analysis, respectively.

**Figure 10:**
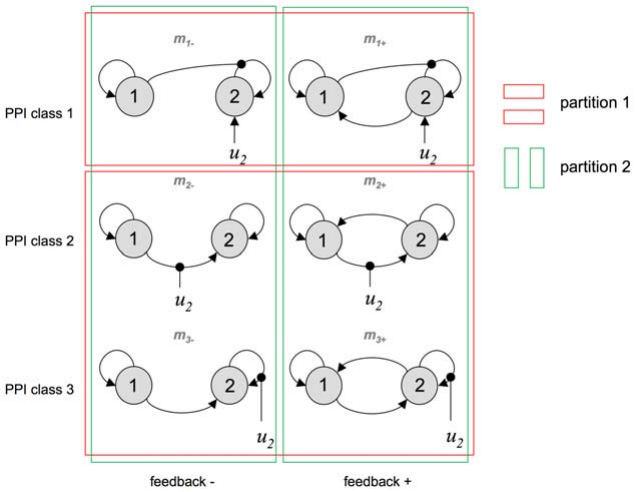
PPI: the 3×2 factorial DCM comparison set. The figure depicts the set of DCMs that are compatible with a PPI (correlation between region 2 and the interaction of region 1 and manipulation 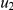). This comparison set is constructed in a factorial way: (i) three PPI classes and (ii) with/without a feedback connection from node 2 to node 1. It can be partitioned into two partitions of two families each. Partition 1 corresponds to the two qualitatively different interpretations of a PPI (“region 1 modulates the response of region 2 to 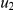
” versus “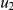 modulates the influence of region 1 onto region 2”). Partition 2 relates to the presence versus absence of the feedback connection.

In the first simulation, we examined how different interpretations of a PPI could be disambiguated by comparing DCMs. One demonstrates a PPI by showing that the activity in region 2 can be explained by the interaction between the activity of region 1 and a psychological factor 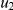 [26]–[27]. There are two qualitatively different interpretations of such effects: either region 1 modulates the response of region 2 to 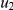, or 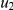 modulates the influence region 1 exerts on region 2. A standard activation analysis of PPI cannot disambiguate these interpretations. However, they correspond to different DCMs. Figure 10 depicts six DCMs that are compatible with the same PPI. This is a 3×2 factorial model comparison set, with the following factors (see Table 1):

- *Class of PPI*. A DCM compatible with the notion that region 1 modulates the region 2 response to 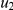 would be such that 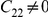 and 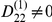 (model 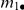). In contradistinction, one could think of at least two DCMs compatible with 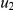 modulating the influence of region 1 onto region 2: 
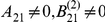
 and 
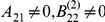
 (models 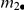 and 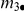, respectively).
- *Presence of a feedback connection*. In addition, one could include or omit a feedback connection from region 2 to region 1. We will denote 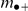 models with such a feedback (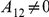) and 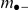 without (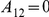).

**Table 1:** 3×2 factorial comparison set for PPI.

	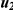 modulates 1→2		1 modulates 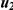 →2
	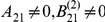	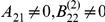	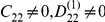
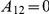 (no feedback)	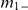	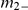	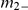
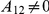 (feedback)	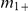	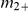	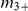

**Figure 11:**
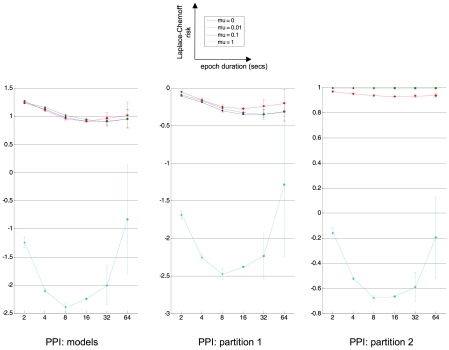
PPI: optimal epoch duration. This figure shows plots of the average (across jitters) Laplace-Chernoff risk as a function of epoch duration (in seconds) and prior expectation 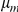 of neural evolution parameters, for the three inference levels defined in relation to the PPI comparison set of Fig. 10. It uses the same format as Fig. 8. Left: model comparison, middle: family comparison (partition 1), right: family comparison (partition 2).

We first ask whether we can find the optimal epoch duration that discriminates among the PPI comparison set, either at the model level or at the family level [23]. We considered two partitions of the comparison set (see Figure 10): (i) partition 1 separates the two qualitatively different interpretations of PPIs and (ii) partition 2 separates models with and without feedback connections. We then adapted the analysis of section “Laplace-Chernoff risk for canonical network identification questions”, as follows:

We considered blocked on/off (square wave) designs, and varied the epoch duration within the range 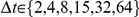. In addition, we varied the first-order moment of the prior densities over evolution parameters 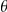 within the range 
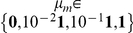
, where 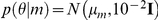. In other respects, the simulation parameters were as above. For all stimulation paradigms, the fMRI session was assumed to last for five minutes. Note that the experimental designs that were balanced in terms of the number of repetitions of factorial conditions (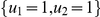, 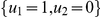, 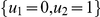 and 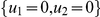). Figure 11 depicts the average (across random jitters) Laplace-Chernoff risk as a function of both epoch duration and the prior mean of the evolution parameters, for the three comparisons, i.e. at the model level and for the two above partitions.

One can see that for strong coupling strengths, the optimal block length seems to be about 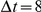 seconds, irrespective of the level of inference. Note that this is slightly smaller than the optimal block length in activation studies [10]. In addition, one can see that the level of inference impacts upon the absolute Laplace-Chernoff risk. For example, it is easier to discriminate between the two qualitative interpretations of the PPI (i.e., family level inference, between the two subsets of partition 1), than to perform an inference at the model level. Interestingly, the most risky inference is about the presence of feedback connections, which reproduces the results in section “Laplace-Chernoff risk for canonical network identification questions”.

In a second simulation, we demonstrate how the Laplace-Chernoff risk could be optimized with respect to the use of TMS. More precisely, we addressed the question of choosing the intervention site, i.e. either on region 1 or on region 2. This defines three possible designs: TMS1 (intervenes on region 1), TMS2 (intervenes on region 2) and no TMS.

**Figure 12:**
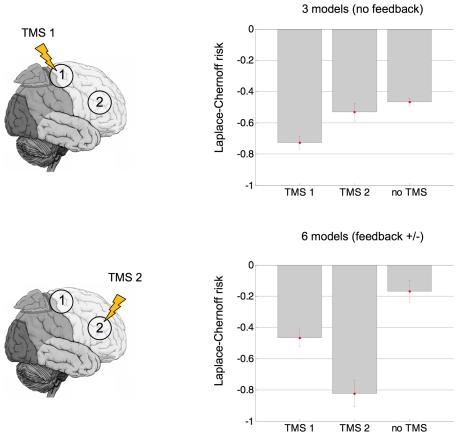
PPI: optimal TMS intervention site. This figure shows plots of the average (across jitters) Laplace-Chernoff risk as a function of the TMS design (TMS1, TMS 2 or no TMS), for two different PPI comparison sets. Left: the two TMS ‘on’ designs (TMS1: target region 1, TMS2: target region 2). Upper-right: average Laplace-Chernoff risk for the first family of partition 2 (three models, no feedback connection from node 2 to node 1). Lower-right: average Laplace-Chernoff risk for the whole PPI comparison set (six models, with and without a feedback connection from node 2 to node 1).

We assumed TMS was used ‘on-line’, using brief stimulation pulses grouped in epochs of 8 seconds duration. We used balanced on/off designs and 5 minutes scanning sessions. To distinguish the physiological effect of TMS from other experimental stimuli, we chose prior densities on evolution parameters that emulated comparatively weak effects; i.e., 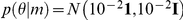. Priors on the observation parameters and the precision hyperparameter were set as above. We draw 16 samples with different random jitters (standard deviation: 2 seconds). Figure 12 depicts the average Laplace-Chernoff risk for the three TMS designs, for two comparison sets: (i) the first subset of partition 2 (only the models without feedback) and (ii) the full comparison set (with and without feedback connections).

One can see that using on-line TMS generally improves the discriminability over models, irrespective of the comparison set (the Laplace-Chernoff risk of the ‘no TMS’ design is systematically higher than those of ‘TMS1’ and ‘TMS2’). However, the optimal intervention site (region 1 or region 2) does depend upon the comparison set: one should stimulate region 1 if one is only interested into discriminating between the ‘no-feedback’ models, and region 2 if one wants to select the best among all models. This makes intuitive sense, since stimulating region 2 (orthogonally to the other experimental manipulations 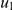 and 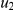) will disclose the presence of the feedback connection more readily.

### Empirical validation

**Figure 13:**
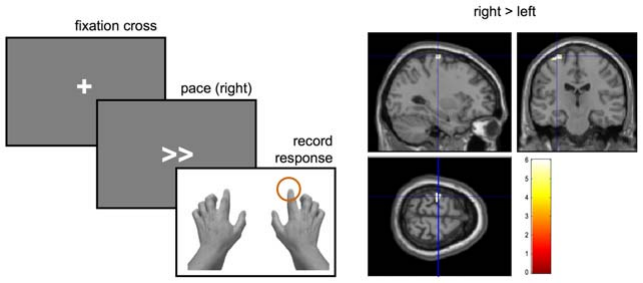
Finger-tapping task: paradigm and classical SPM. Left: inner stimulation sequence of one trial of the finger-tapping task (fixation cross, then motor pacing – left or right or both- and the final recording of the subject's response – button press-). Right: SPM t-contrast (right>left) thresholded at p = 0.05 (FWE corrected) for subject KER under the blocked design.

In this section, we apply the above approach to empirical fMRI data acquired during a simple finger-tapping (motor) task. Figure 13 reports the structure of the task.

Each trial consisted of a fixation period and a pacing stimulus (‘right’, ‘left’, ‘right and left’ or null) that ended with the subject's motor response (button press). The whole fMRI session comprised 400 events (100 left, 100 right, 100 left & right, 100 null events). The average inter-trial interval was two seconds. Each subject participated in two sessions, corresponding to two variants of the experimental design, i.e., blocked (ten consecutive identical trials per block) and event-related (randomized trials). There were two subjects in total (but see above).

About 700 T2*-weighted single-shot gradient echo echo-planar images (TE = 40 ms, TR = 1.3 s, 24 interleaved axial slices of 4.4 mm thickness, FOV = 24×24 cm^2^, 80×80 matrix) were acquired over a 35-min session on a 3 Tesla MRI scanner. FMRI data were pre-processed using SPM8 (http://www.fil.ion.ucl.ac.uk/spm/). EPI time series were realigned, spatially smoothed with an 8 mm FWHM isotropic Gaussian kernel and normalized. A GLM was constructed to assess the presence of regional BOLD changes related to the motor responses. The design matrix contained two pacing regressors (‘left’ and ‘right’), as well as realignment parameters to correct for motion-related changes.

Left and right motor cortices (MC) were identified by means of subject-specific *t*-contrasts testing for the difference between the ‘left’ and ‘right’ pacing conditions (p<0.05, whole-brain FWE corrected, see Figure 13). A summary time series was derived for each ROI by computing the first eigenvariate of all suprathreshold voxel time series within 10 mm of the ROI centres.

**Figure 14:**
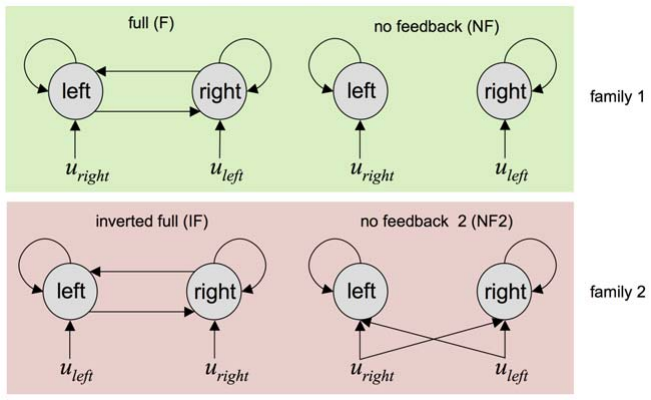
Finger-tapping task: DCM comparison set. The figure depicts the DCM comparison set we used to analyze the finger-tapping task fMRI data. This set can be partitioned into two families of models. Family 1 gathers two plausible network structures for the finger-tapping task (left pace drives right motor cortex and right pace drives left motor cortex, with and without feedback connections). Family 2 pools over two implausible motor networks subtending the finger-tapping task (allowing the left pace to drive the left motor cortex, and reciprocally).

Four models were included in the comparison set, which is depicted in Figure 14:

- Full model (F): the left (respectively, right) MC is driven by the ‘right’ (respectively, ‘left’) pace. Feedback connections between both MC are included.
- Inverted full (F): the driving effects of the pacing stimuli are inverted, when compared to model F.
- No feedback (NF): similar to F, but without the feedback connections.
- No feedback 2 (NF2): each pacing stimulus is allowed to drive both motor cortices.

We know that motor action is associated with activity in the contralateral motor cortex. This establishes a point of reference for our model comparisons (akin to the “ground truth” scenario used for validating models by simulated data). We therefore assume that models F or NF best capture the motor preparation processes during the finger-tapping task. We will thus place the inference at the family level, with two families: (i) family 1: models F and NF and (ii) family 2: models IF and NF2. A selection error thus arises whenever the posterior family comparison selects family 2.

We can now derive the Laplace-Chernoff risk for the two designs (blocked versus event-related). This is summarized in Table 2 above, as a function of the first-order moment of the prior densities over neural evolution parameters 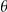 within the range 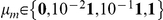, where 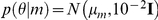. As in the simulations, we used i.i.d. shrinkage priors for the hemodynamic evolution and observation parameters (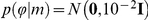) and the expected noise precision was 0.05.

**Table 2:** Laplace-Chernoff risks for the event-related versus blocked design (when comparing family 1 versus family 2).

	event-related design	blocked design
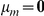	−1.26	−1.63
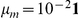	−1.21	−1.61
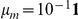	−0.92	−1.70
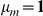	−0.96	−3.74

One can see that the Laplace-Chernoff risk is smaller for the blocked-design than for the event-related design, irrespective of the first-order moment 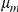 of the neural evolution parameters prior density. In addition, it seems that the event-related design is much less sensitive to a change in 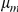 than the blocked design.

**Figure 15:**
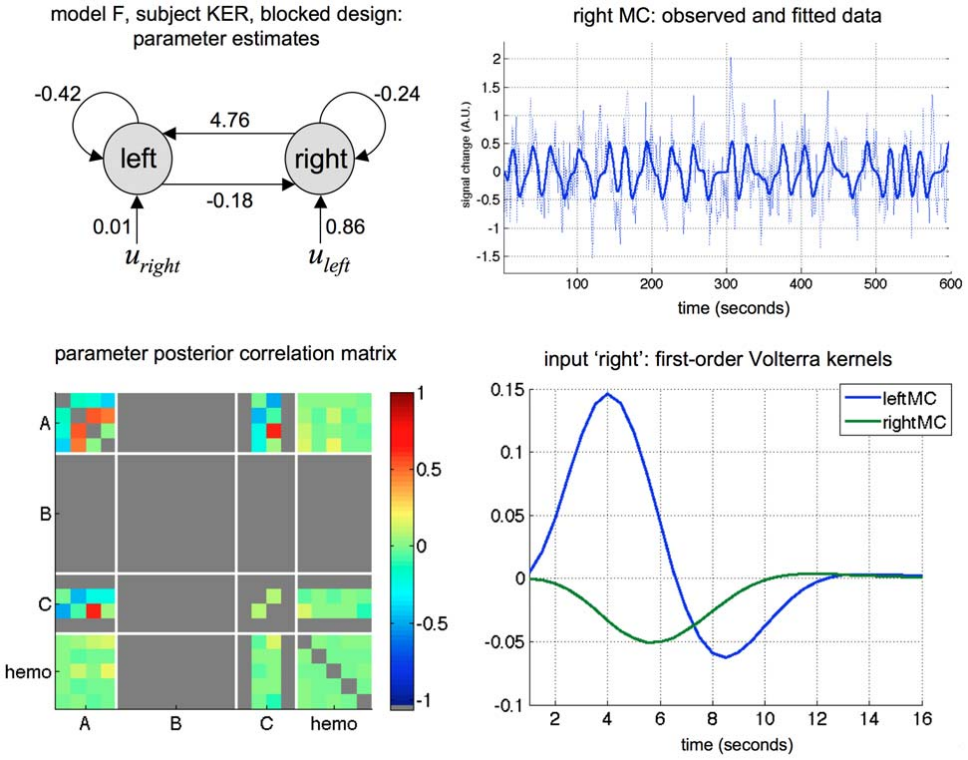
Finger-tapping task: VB inversion of model F under the blocked design (subject KER). Upper-left: estimated coupling strengths of model F, under the blocked design (subject KER). These are taken from the first-order moment of the approximate posterior density over evolution parameters. Lower-left: parameter posterior correlation matrix. Upper-right: observed versus fitted data in the right motor cortex. Lower-right: linearised impulse responses (first-order Volterra kernels) to the ‘right’ pace in both motor cortices as a function of time.

We then inverted the four models using the variational Bayesian approach under standard shrinkage priors (see section “Laplace-Chernoff risk for canonical network identification questions” above), for both subjects and both designs. Figure 15 summarizes the inversion of model F for subject KER, under the blocked design.

**Figure 16:**
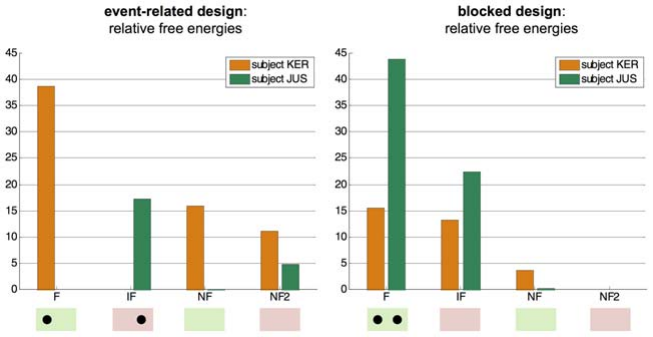
Finger-tapping task: DCM comparison results. This figure plots the log-model evidences of the four DCMs included in the comparison set for both subjects (orange bars: subject KER, green bars: subject JUS) and both designs (left: event-related, right: blocked design). Green (respectively, rose) shaded areas indicate the models belonging to family 1 (respectively, family 2). Black dots show the four winning models (one per subject and per design). Note that the free energies are relative to the minimal free energy within the comparison set, for each subject and design.

One can see that the observed BOLD responses are well fitted by the model. Not surprisingly, inspection of the first-order Volterra kernels [28] shows that the average response of the left MC to the ‘right’ pacing stimuli is positive and bigger in amplitude than that of the right MC (and reciprocally). Also, there are very small posterior correlations between the hemodynamic and the neuronal parameters, which reflect their identifiability. However, further inspection of the posterior correlation matrix shows that, for this particular dataset and model, the feedback connections and the driving effects of the pacing stimuli are not perfectly separable. This means that the design is not optimal for a precise estimation of these parameters. However, one can still compare the two designs in terms of how well they can discriminate the four DCMs included in the comparison set. This is summarized in Figure 16, which plots the free energies of the four models, for both subjects and both designs.

One can see that no model selection error was made under the blocked design, whereas there was one model selection error for subject JUS under the event-related design. Deriving the posterior probabilities of model families shows exactly the same result. Thus, as predicted by the Laplace-Chernoff risk (c.f. Table 2), the observed error selection rate is higher for the event-related design than for the blocked design.

**Figure 17:**
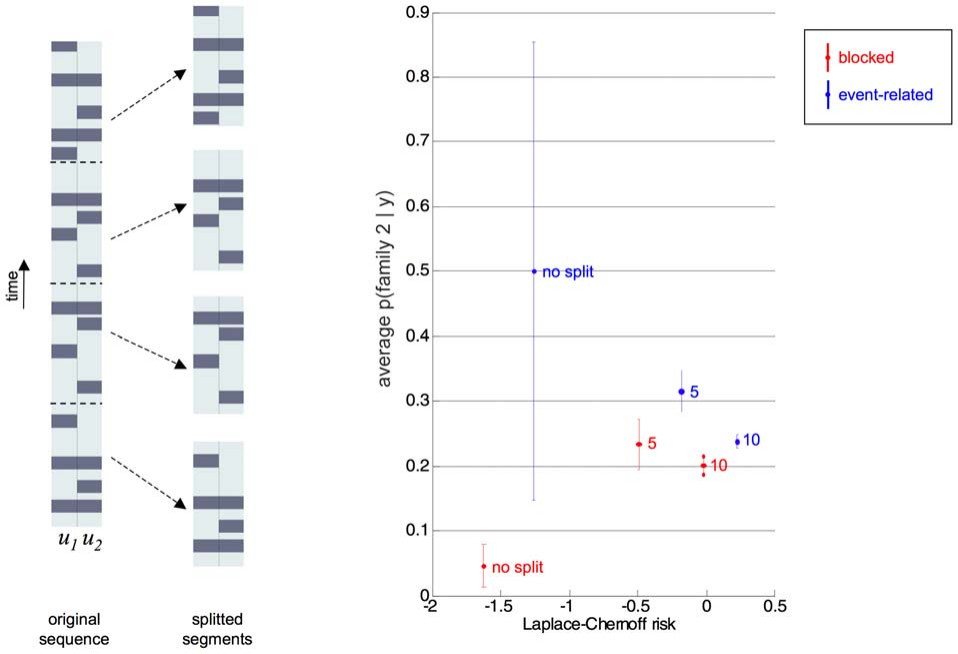
Finger-tapping task: splitting analysis. This figures summarizes the results of the splitting analysis (see main text), in terms of the relationship between the Laplace-Chernoff risk and the observed model selection error rate. Left: splitting procedure. The complete data and input sequence (one per subject and per design) is split into 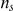 segments, each of which is analyzed independently. Right: the average (across segments and subjects) probability of making a model selection mistake (i.e. 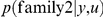) is plotted as a function of the Laplace-Chernoff risk, for both designs (blue: event-related, red: blocked). Each point corresponds to a different splitting procedure (no split, split into 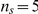 segments, split into 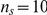 segments).

One may wonder how reliable this result is, given that only two subjects were used to derive the selection error rate. This is because a solid validation of the Laplace-Chernoff risk necessitates an estimate of the model selection error rate in terms of the frequency of incorrect model selections (as in section “Evaluation of the model selection error bounds”). We thus performed the following analysis:

For each subject and each design, we first split the data (and the stimulation sequence) into 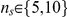 consecutive segments (see Figure 17). This allows us to artificially inflate the number of subjects (by five and ten, respectively), at the cost of reducing the effective sample size for each ‘subject’. We can then derive the Laplace-Chernoff risks for the splitting procedure, i.e.: (i) no split (as above), (ii) split into 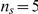 segments and (iii) split into 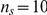 segments. In addition, we can conduct a complete analysis for each segment independently of each other; i.e., invert the four DCMs included in the comparison set, derive the posterior probabilities over model families, and perform the comparison. The cost of this procedure is a loss of total degrees of freedom (and thus model discriminability power), since we allow the model parameters to vary between each data segment. However, this allows us to artificially increase the number of model selections, by considering each segment as a dummy subject. Note that the posterior probability of family 2 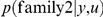 measures the objective probability of making a model selection error (see Equation 6). Averaging 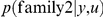 across segments and subjects thus provides an approximation to the true selection error rate under both designs (see Equation 7). This serves as sampled reference for the Laplace-Chernoff risk. Figure 17 summarizes the results of this analysis.

First, one can see that the Laplace-Chernoff risk of the blocked design is always smaller than that of the event-related design, irrespective of the number of splits. Second, this difference decreases as the number of splits increases. The average selection error rate exactly reproduces this pattern. First, the observed error rate is higher for the event-related design than for the blocked design, irrespective of the number of splits. Second, this difference decreases as the number of splits increases. However, in this example, the Laplace-Chernoff risks increases as the number of splits increases, irrespective of the particular design used. This is in contradiction with the observed selection error rate, which seems to increase as the number of splits increases, only for the blocked design (as opposed to the event-related design). This might be due to a different optimal balance between number of subjects and sample size per subject for the two designs. We will comment on these issues in the discussion. Nevertheless, this splitting procedure provides further evidence that the Laplace-Chernoff risk is a reliable predictor of the average selection error rate, and hence a useful metric for comparing experimental designs.

## Discussion

In this article, we have proposed a general method for optimizing the experimental design to maximise the sensitivity of subsequent Bayesian model selection. We have examined design optimization in the specific context of effective connectivity methods for fMRI and have focused on how to best decide among hypotheses about network structure and the contextual modulation of specific connections therein. We reiterate, however, that our method is very general and is applicable to any generative model of observed data (e.g., brain activity or behavioural responses, c.f., e.g., [29]).

Our method relies upon the definition of a statistical risk, in terms of an approximate information theoretic bound on the model selection error rate. Theoretical and numerical evaluations of the proposed Laplace-Chernoff risk demonstrate its reliability. This optimality criterion was then applied to the problem of optimising design when identifying the structure of brain networks using DCM for fMRI data. Using both numerical evaluations and empirical fMRI data, we examined the impact of the priors (on model parameters), the level of inference (model versus family) and the specific question about network structure (the model comparison set) on the optimal experimental design. For example, we have shown that asking whether a feedback connection exists requires shorter epoch durations than when asking whether there is a contextual modulation of a feedforward connection. In addition, our empirical results suggest that the method has good predictive validity (as established with the splitting analysis). In the following, we discuss the strengths and limitations of the approach as well as potential extensions.

First, one may wonder how general the proposed design optimality criterion for (Bayesian) model comparison is. In other words, one could start from a completely different perspective and ask whether it would be possible to derive another design optimality criterion that would eventually yield another optimal design for the same model comparison set. A first response to this question draws on the equivalence with the classical design efficiency (c.f. section “Tightness of the Laplace-Chernoff bounds”), which shows that in specific circumstances (flat priors, nested linear models); the Laplace-Chernoff risk is monotonically related to frequentist statistical power. We conjecture this to be a very general statement that applies whenever Bayesian model comparison can be reduced to classical hypothesis testing (in the frequentist limit). This is important, since it means that the Laplace-Chernoff optimal design would be no different from established classical designs. Interestingly, it seems that the use of the Jensen-Shannon divergence 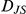 for design optimality can be justified from purely information theoretic considerations, without reference to the model selection error rate [30]–[31]. The degree to which the two approaches are similar (and/or generalize other schemes such as classical design efficiency) will be the focus of subsequent publications, in collaboration with these authors (evidence in favour of the equivalence between the two frameworks arose from a very recent informal meeting with Dr. A. G. Busetto, who independently derived his own approach). In our opinion, the most relevant line of work, in this context, is to finesse the necessary approximations to the Jensen-Shannon divergence. This is because different approximations to the Chernoff bound could lead to different approximate optimal designs. We will discuss this particular issue below.

The numerical simulations we have conducted identified general factors that have an unambiguous influence on design efficiency, namely: the number of models and the data dimension (see section “Tightness of the Laplace-Chernoff bounds”), as well as the signal-to-noise ratio (SNR, see section “Evaluation of the model selection error bounds”). Note that increasing the data dimension enables two (or more) models to make distinct predictions, provided that their respective predictive densities differ sufficiently (c.f. Figure 3). This is because uncontrolled variability in the data can be averaged out. In other terms, increasing the data dimension simply increases the effective SNR. In summary, the overall discriminative power of any design increases with the effective SNR, and decreases with the number of models. Both the effective SNR and the typical number of models will usually depend upon the modelling context. We have presented numerical simulations (and empirical data analyses) that span the realistic range of the effective SNR, when analyzing fMRI data with DCM. Typically, one would focus on a set of two to five regions of interest, with fifteen minutes session duration (i.e., for typical fMRI sampling rates, the data dimension is of order 10^3^). The SNR may depend upon the anatomical location of the network (e.g., lower SNR for subcortical compared to cortical structures), but should be of the order of 1 dB. In terms of the size of the comparison set, we have deliberately chosen to keep this small; although it can vary from one study to the next, depending upon network dimensionality and prior knowledge. However, we anticipate that hypothesis-driven experiments that would benefit from design optimization will focus on the comparison of a handful of models or families of models (see Text S3). In other words it may be difficult to design a study that can discriminate efficiently among a few thousands of models (or more). This is because of the inevitable dilution of experimental evidence across models (see, e.g., [23]). Recall that the exact probability of making a model selection error can be evaluated a posteriori, following Equation 6. Typically, the winning model among a few thousand alternatives will never attain a posterior probability of about 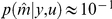, which leads to an unacceptable model selection error probability of at least 0.9!

Second, one may ask whether the Laplace-Chernoff risk is a suitable criterion for choosing among potential designs within the context of a group analysis. This is because we did not consider a (more general) hierarchical scenario, which would account explicitly for the variability of the hidden model within a group of subjects (i.e., random effects analysis [32]). In this case, the total variability consists of within- and between-subject sources of variation. So far, our approach consists of optimizing the experimental design by controlling the variability at the within-subject level. This is done by optimizing the discriminability of models included in the comparison set. In essence, this is similar to design optimization for classical GLM analyses, where optimality is defined in relation to the reliability of maximum likelihood estimators. In this context, one can find an optimal balance between the number of subjects and the sampling size per subject [33]. This balance strives for a principled way of choosing, for example, between a study with twenty subjects scanned for fifteen minutes each versus a study with ten subjects scanned for half an hour each. In [34], authors demonstrate how this balance depends upon the ratio of within- and between- subject variances. Our analysis of the empirical data seems to disclose a similar dependency (Figure 17). In brief, the relationship between the average error rate and the sharing of degrees of freedom (across the within- and between-subject levels) depends upon the design type (i.e. blocked versus event-related). The results in sections “Laplace-Chernoff risk for canonical network identification questions” and “Investigating psycho-physiological interactions with DCM” imply that it may depend upon the comparison set as well. In addition, one has to consider two sorts of random effects here: variability in the model parameters (for a fixed model), and variability in the hidden model itself. Future work will consider these issues when extending the present approach to a multi-level random effects analysis for group data.

Third, the Laplace-Chernoff bound relies upon the derivation of the prior predictive density of each model included in the comparison set. For nonlinear models, it relies upon a local linearization around the prior mean of the parameters; similarly to classical procedures for design optimization (see, e.g., [35] for an application to estimating the hemodynamic response function). We are currently evaluating the potential benefit of using variants of the unscented transform [22], which may yield a more accurate approximation to the prior predictive density. We have not, however, accounted for uncertainty on hyperparameters; e.g., moments of the prior density on noise precision. Note that we do not expect this to be crucial because the contribution of the prior uncertainty on these hyperparameters is negligible, when compared to the variability already induced in the prior predictive densities.

Nevertheless, the above approximations induce potential limitations for the current approach. For example, numerical simulations in sections “Tightness of the Laplace-Chernoff bounds” and “Results” demonstrate that the Laplace approximation might cause the bound to “break”, i.e. the Laplace-Chernoff risk might become an over-optimistic estimate of the model selection error rate. More precisely, this happens in situations where the exact model selection error rate is already very low (typically below 0.2, see Figure 3). Having said this, the relationship between the Laplace-Chernoff risk and the exact model selection error rate always remained monotonic. This means that the design that minimizes the Laplace-Chernoff risk is the one that would have minimized the exact model selection error rate, had we been able to quantify it. This monotonic relationship remains to be empirically verified for classes of models that are more complex than DCMs.

From a practical perspective, if the aim is to quantify the actual model selection error rate (or a conservative upper bound on it), then the Laplace-Chernoff risk will yield an accurate estimation only for poorly discriminative designs (importantly, the upper bound on the true model selection error rate becomes tightest for the least decisive model comparisons, i.e., the approximation by the Laplace-Chernoff risk is most accurate when it is most needed). However, in most practical applications the aim is simply to select the most discriminative design amongst several alternatives. In this case, the Laplace-Chernoff risk can be used for any model comparison.

Fourth, one may consider other applications for the Laplace-Chernoff risk. For example, given an experiment whose design is fixed or cannot be specified *a priori* (e.g., the presence of epileptic spikes, or successful vs. failed retrieval of encoded memories), one can use our approach to distinguish between statistical questions for which the design is suitable and those for which they are not. This can be done by evaluating the Laplace-Chernoff risk for different comparison sets or partitions of the same comparison sets. This could also be useful to motivate the *a priori* pruning of competing hypotheses in a principled way. One could also think of using an adaptive design strategy where the paradigm is optimized online as the experiment progresses (see [36]–[37] for similar applications to fMRI). Even though such procedures will not lead to a major gain in efficiency for linear models, this can be quite different for nonlinear models of the sort employed in DCM [38]. This is because the progressive accumulation of information corrects the predictive densities that are required to compute the Laplace-Chernoff risk. In turn, this can be exploited to improve the overall model discriminability [39].

Fifth, we would like to highlight some important properties of the biophysical models when optimizing the experimental design for identifying networks with DCM for fMRI data. Consider the increase in selection error rate at short epoch durations. This is likely to arise from the hemodynamic impulse response function, which induces strong correlations in the fMRI data at fast time scales, relative to its own (about 16 to 32 seconds). Such loss of discriminative power in high frequencies has been discussed in the context of design optimization for classical fMRI studies [12]. This effect worsens at very short epoch durations, due to *hemodynamic refractoriness*; i.e., the response to a second stimulus is reduced if it follows the preceding stimulus with a short delay [40]. This saturation effect is known to be captured by the hemodynamic Balloon model that is part of DCM [28]. Interestingly, the effect of these known phenomena on statistical efficiency depends on which particular scientific question is asked. For example, the identification of feedback connections within the network is facilitated by epoch durations that are much shorter than required for addressing other questions about effective connectivity or in conventional GLM analyses (cf. Figure 8). This is because a feedback connection expresses itself mainly when the system goes back to steady-state, through an asymmetrical increase in node-to-node correlation (cf. Figure 9). In other terms, a feedback connection manifests itself by a higher reproducibility of network decay dynamics across repetitions, which is why its detection requires short epoch durations and thus a more frequent repetition of the transient that discloses its effect on the data.

Sixth, our preliminary results show that the use of interventional techniques such as TMS could be highly beneficial for reducing the selection error rate (Figure 12). However, the expected gain is strongly dependent upon its physiological effects, which are still not fully known [41]. For example, different stimulation frequencies target different populations of neurons and can therefore either have a net excitatory or inhibitory effect. Such effects can be modelled easily within the framework of DCM [42] and would constitute a straightforward extension to the example given in this paper (see [43] for related work). In the future, such extensions could allow one to ask which TMS technique one should use to maximally improve sensitivity in disclosing network mechanisms by model selection. Such combinations of experimental techniques and model-based analysis are starting to emerge in the field [44] and hold great promises for the identification of directed influences in the brain, provided that one understands the impact of the experimental design used.

Lastly, numerical simulations showed that the optimal design depends upon the choice of priors on the model's parameters 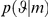. This is of course expected, because 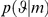 partly determines the model's prior predictive density over data 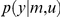 (c.f. equations 1–2). Strictly speaking, we cannot use noninformative priors when optimizing the design for model comparison. This is because, in most cases, this would induce flat prior predictive densities for all models, which would prevent any design optimization procedure. This means that we have to choose mildly informative priors for the model's parameters. However, the precise way in which the priors affect the efficiency of the design depends upon the comparison set. For example, increasing 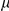 (the prior mean over the connectivity parameters) either increases model discriminability (e.g., Figure 10, for the feedback/no feedback comparison) or decreases it (e.g., Figure 10, when deciding where the input enters the network). Recall that a (generative) model is defined by all the (probabilistic) assumptions that describe how the data are generated, including the prior 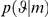. This means that when using different values for 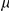, we are effectively defining different models. Thus, varying both 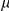 and the connectivity structure implicitly augments the comparison set in a factorial way. Assuming that one is only interested in selecting the connectivity structure (irrespective of 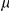), one has to resort to family inference (see Text S3), where each family is composed of members that share the same connectivity structure but differ in their 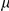. This simply means deriving the Laplace-Chernoff risk after marginalizing over 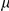. This basically treats 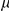 as a nuisance effect, and de-sensitizes the design parameter of interest to mathematical variations in the implementation of the model. We have shown examples of such a “family level” extension of optimal designs when inspecting canonical PPI models (section “Investigating psycho-physiological interactions with DCM”) and analyzing experimental data (section “Empirical validation”).

Similarly, one might wonder how sensitive the optimal design is to variations of the neuronal and biophysical state equations used in the DCM framework. Preliminary results (not shown here) indicate that the effects of design parameters such as epoch duration are not very sensitive to such variations, e.g., two-state DCM [42] or stochastic DCM [45]–[46]. However, the latter class of DCM asks for a slight modification in the derivation of the prior predictive density [47]. This is because the presence of neural noise induces additional variability at the level of hidden states. Typically, neural noise expresses itself through a decrease in lagged (intra- and inter-node) covariances. This might therefore induce noticeable changes in optimal design parameters for specific comparison sets. A general solution to this is to include the DCM variant as a factor in the model comparison set, and then again, use family level inference to marginalize over it.

We envisage that the present approach will be useful for a wide range of practical applications in neuroimaging and beyond. It may be particularly helpful in a clinical context, where the ability to disambiguate alternative diseases mechanisms with high sensitivity is of great diagnostic importance. One particular application domain we have in mind for future studies concerns the classification of patients from spectrum diseases such as schizophrenia using mechanistically interpretable models [48]. Another potential future application concerns model-based prediction of individual treatment responses, based on experimentally elicited physiological responses (e.g., to pharmacological challenges [49]). Either approach will greatly benefit from methods for optimizing experimental design, such as the one introduced here.

### Software note

All the routines and ideas described in this paper will be implemented in the academic freeware SPM (http://www.fil.ion.ucl.ac.uk/spm).

## Supporting Information

Text S1 **The Laplace approximation to the Jensen-Shannon bound.**
(DOCX)

Text S2 **The Laplace-Chernoff risk for the general linear model and its frequentist limit.**
(DOCX)

Text S3 **Extension to the comparison of model families.**
(DOCX)
