# Supplementary material for: Optimizing Experimental Design for Comparing Models of Brain Function
Source: PLoS Comput Biol. 2011 Nov 17;7(11):e1002280. doi: 10.1371/journal.pcbi.1002280 (PMC3219623; doi:10.1371/journal.pcbi.1002280)
Supplement: Text S1 — The Laplace approximation to the Jensen-Shannon bound. (DOCX) [file pcbi.1002280.s001.docx]

**Optimizing experimental design for comparing models of brain function**

**Appendix 1: The Laplace approximation to the Jensen-Shannon bound**

In this section, we will derive an analytical approximation to the Jensen-Shannon bound for the class of generative models that have the following form:

$y=g_{m}\left(\vartheta,u\right)+\varepsilon$, A1.1

where $g_{m}$ is the deterministic (observation) mapping of model $m$, $u$ is some design parameter and $\varepsilon$ is a residual (additive measurement) error. Without loss of generality (under appropriate nonlinear transformations), the likelihood and prior densities are:

$m:\left\{\begin{matrix} p\left(y\left|\vartheta,m,u\right.\right)=N\left(g_{m}\left(\vartheta,u\right),Q_{m}\right) \\ p\left(\vartheta\left|m\right.\right)=N\left(\mu_{m},R_{m}\right) \end{matrix}\right.$, A1.2

where $Q_{m}$ is the covariance matrix of $\varepsilon$ and $\left(\mu_{m},R_{m}\right)$ are the prior mean and covariance of the unknown parameters $\vartheta$ (under model $m$).

For this class of models, the Jensen-Shannon divergence could still be difficult to compute because of the arbitrary complexity of the ensuing marginal likelihoods. The integration in equation 1 can then be finessed by using a first-order Taylor expansion to the observation function around the prior mean $\mu_{m}$ of the unknown parameters:

$\begin{matrix} p\left(y\left|m,u\right.\right)\approx N\left(g_{m}\left(\mu_{m},u\right),\tilde{Q}_{m}\left(u\right)\right) \\ \tilde{Q}_{m}\left(u\right)=Q_{m}+\left.\frac{\partial g_{m}}{\partial\vartheta}\right|_{\mu}R_{m}\left.\frac{\partial g_{m}}{\partial\vartheta}\right|_{\mu}^{T} \end{matrix}$. A1.3

We then use a further Gaussian (moment-matching) approximation to the marginal density $p\left(y\left|u\right.\right)$, which, following equations 3 and A1.3, has the form of a mixture of Gaussians:

$\begin{matrix} p\left(y\left|u\right.\right)\approx N\left(\mu_{y}\left(u\right),V_{y}\left(u\right)\right) \\ \mu_{y}\left(u\right)=\sum_{m\in M} p\left(m\right)g_{m}\left(\mu_{m},u\right) \\ V_{y}\left(u\right)=\sum_{m\in M} p\left(m\right)\left(\left(g_{m}\left(\mu_{m},u\right)-\mu_{y}\left(u\right)\right)\left(g_{m}\left(\mu_{m},u\right)-\mu_{y}\left(u\right)\right)^{T}+\tilde{Q}_{m}\left(u\right)\right) \end{matrix}$ A1.4

Equations A1.3 and A1.14 yield an analytical “Laplace” approximation $-r\left(u\right)$ to the Jensen-Shannon divergence (as given in equation 9), which we propose as a proxy for the original Bayesian design risk:

$\begin{matrix} D_{JS}\left(u\right)\approx-r\left(u\right) \\ r\left(u\right)=\frac{1}{2}\left(\sum_{m\in M} p\left(m\right)\log\left|\tilde{Q}_{m}\left(u\right)\right|-\log\left|V_{y}\left(u\right)\right|\right) \\ =\frac{1}{2}\left(\sum_{m\in M} p\left(m\right)\log\left|\tilde{Q}_{m}\left(u\right)\right|-\log\left|\sum_{m\in M} p\left(m\right)\left(\Delta g_{m}\Delta g_{m}^{T}+\tilde{Q}_{m}\left(u\right)\right)\right|\right) \\ \Delta g_{m}=g_{m}\left(\mu_{m},u\right)-\mu_{y}\left(u\right) \end{matrix}$ A1.5

Together with equation 8, equation A1.5 allows us to approximate both the lower and the upper Chernoff bound to the model selection error rate $p\left(\hat{e}=1\left|u\right.\right)$. Let the *Laplace-Chernoff* *risk* $b_{LC}\left(u\right)$ be defined as follows:

$b_{LC}\left(u\right)\equiv H\left(p\left(m\right)\right)+r\left(u\right)$ A1.6

where $r\left(u\right)$ is the Laplace approximation to the Jensen-Shannon divergence (c.f. Equation A1.5). In brief, the Laplace-Chernoff risk is the simplest possible analytical approximation to the Chernoff bound that explicitly depends upon the two first moments of the prior predictive densities $p\left(y\left|m,u\right.\right)$.
