# Supplementary material for: Optimizing Experimental Design for Comparing Models of Brain Function
Source: PLoS Comput Biol. 2011 Nov 17;7(11):e1002280. doi: 10.1371/journal.pcbi.1002280 (PMC3219623; doi:10.1371/journal.pcbi.1002280)
Supplement: Text S2 — The Laplace-Chernoff risk for the general linear model and its frequentist limit. (DOCX) [file pcbi.1002280.s002.docx]

**Optimizing experimental design for comparing models of brain function**

**Appendix 2: Laplace-Chernoff risk for the general linear model and its frequentist limit**

In this section, we derive the closed form expression of the design risk associated with comparing the null ($H_{0}:\vartheta_{i}=0$) with the alternative hypothesis $H_{1}:\vartheta_{i}\neq0$. Within a general linear model of the form given in Equation 18, this is equivalent to comparing two models:

- $m_{1}$: a GLM with the full design matrix $X$,
- $m_{0}$: a GLM with a reduced design matrix $X_{\backslash i}$, i.e. without its *i-*th column $X_{i}$,

where we have dropped the dependence of the design matrix upon the design $u$ (i.e.: $X\equiv X\left(u\right)$) for notational simplicity.

Under i.i.d. Gaussian priors for the unknown parameters $\vartheta$ and residuals $\varepsilon$ (i.e. $Q_{m}=\sigma^{2}I$ and $R_{m}=\alpha^{2}I$), their respective prior predictive densities are given by:

$\left\{\begin{matrix} p\left(y\left|m_{0}\right.\right)=N\left(0,\tilde{Q}_{0}\right) : \tilde{Q}_{0}=\sigma^{2}I+\alpha^{2}X_{\backslash i}X_{\backslash i}^{T} \\ p\left(y\left|m_{1}\right.\right)=N\left(0,\tilde{Q}_{1}\right) : \tilde{Q}_{1} =\sigma^{2}I+\alpha^{2}XX^{T}=\tilde{Q}_{0}+\alpha^{2}X_{i}X_{i}^{T} \end{matrix}\right.$ A2.1

Replacing the above expressions for the covariance matrices $\tilde{Q}_{0}$ and $\tilde{Q}_{1}$ in Equations 16 yields the expression for the corresponding Laplace-Chernoff risk:

$b_{LC}\left(u\right)=1-\frac{1}{2}\log\frac{\left|\tilde{Q}_{0}+\frac{1}{2}\alpha^{2}X_{i}X_{i}^{T}\right|}{\sqrt{\left|\tilde{Q}_{0}+\alpha^{2}X_{i}X_{i}^{T}\right|\left|\tilde{Q}_{0}\right|}}$ A2.2

Applying the matrix determinant lemma ($\left|A+uv^{T}\right|=\left|A\right|\left(1+v^{T}A^{-1}u\right)$) gives:

$\begin{matrix} b_{LC}\left(u\right)=1-\frac{1}{2}\log\frac{1+\frac{1}{2}a\left(u\right)}{\sqrt{1+a\left(u\right)}} \\ =1-\frac{1}{4}\log\left(1+\frac{a\left(u\right)^{2}}{4\left(1+a\left(u\right)\right)}\right) \\ \\ a\left(u\right)=\alpha^{2}X_{i}^{T}\tilde{Q}_{0}^{-1}X_{i} \end{matrix}$ A2.3

which is the result summarized in Equation 20 of the main text.

Let us now look at the frequentist limit (non-informative priors, i.e.: $\alpha^{2}/\sigma^{2}\to\infty$) of the optimal design, i.e. the minimiser of the Laplace-Chernoff risk in equation A3. First, note that since $a\left(u\right)>0$ and the function $x\to x^{2}/\left(1+x\right)$ is monotonically increasing on the set of positive real numbers, the optimal design is the design $u*$ that maximizes $a\left(u\right)$:

$\begin{matrix} u*\equiv\mathop{\mathrm{argmin}}\limits_{u} b_{LC}\left(u\right) \\ =\mathop{\mathrm{argmax}}\limits_{u} a\left(u\right) \end{matrix}$, A2.4

by convexity of the $x\to\log x$ mapping. Second, note that the frequentist limit to the inverse prior predictive covariance matrix under the null can be obtained from the Woodbury identity:

A2.5

which is simply proportional to the projection operator onto the null space of the reduced design matrix $X_{\backslash i}$.

Now let us insert equation A5 into equation A3, and compare the ensuing frequentist limit to the expression for the classical design efficiency measure $\varsigma\left(u\right)$ given in equation 19 of the main text. This gives:

$\begin{matrix} \lim_{\alpha^{2}/\sigma^{2}\to\infty} a\left(u\right)=\frac{\alpha^{2}}{\sigma^{2}}X_{i}^{T}\left(I-X_{\backslash i}\left(X_{\backslash i}^{T}X_{\backslash i}\right)^{-1}X_{\backslash i}^{T}\right)X_{i} \\ =\alpha^{2}\varsigma\left(u\right) \end{matrix}$ A2.6

Thus, the optimal design at the frequentist limit is the design that maximizes the classical design efficiency measure:

$\begin{matrix} \lim_{\alpha^{2}/\sigma^{2}\to\infty} u*\equiv\mathop{\mathrm{argmin}}\limits_{u} \lim_{\alpha^{2}/\sigma^{2}\to\infty} b_{LC}\left(u\right) \\ =\mathop{\mathrm{argmax}}\limits_{u} \varsigma\left(u\right) \end{matrix}$ A2.7

which is the result summarized in Equation 20 of the main text.
