# Supplementary material for: Optimizing Experimental Design for Comparing Models of Brain Function
Source: PLoS Comput Biol. 2011 Nov 17;7(11):e1002280. doi: 10.1371/journal.pcbi.1002280 (PMC3219623; doi:10.1371/journal.pcbi.1002280)
Supplement: Text S3 — Extension to the comparison of model families. (DOCX) [file pcbi.1002280.s003.docx]

**Optimizing experimental design for comparing models of brain function**

**Appendix 3: Extension to the comparison of model families**

In this paper, we have mainly considered the problem of selecting a single model from a set of alternatives. However, we may want to compare families of models to address broader questions and reduce uncertainty about the detailed aspects of model structure [23]. Here, we briefly describe how to extend the Laplace-Chernoff risk to the comparison of families of models.

Let $f_{1},f_{2},...,f_{j},...,f_{n_{f}}$ be the subsets of models that specifies a partition of the comparison set, where $n_{f}$ is the number of families in the partition. The Laplace-Chernoff risk $b_{LC}\left(u\right)$ associated with a family selection error is simply derived from the Jensen-Shannon divergence between the prior predictive densities of each of these model families. Let $p\left(y\left|f_{j},u\right.\right)$ be the prior predictive density of model family $f_{j}$. We propose to derive a Gaussian approximation to $p\left(y\left|f_{j},u\right.\right)$ by marginalizing over models belonging to the family $f_{j}$ and then use a moment-matching approach similar to Equation 13:

$\begin{matrix} p\left(y\left|f_{j},u\right.\right)\equiv\frac{1}{p\left(f_{j}\left|u\right.\right)}\sum_{m\in f_{j}} p\left(m\left|u\right.\right)p\left(y\left|m,u\right.\right) \\ \approx N\left(g_{f_{j}}\left(u\right),\tilde{Q}_{f_{j}}\left(u\right)\right) \\ \\ g_{f_{j}}\left(u\right)=\frac{1}{p\left(f_{j}\left|u\right.\right)}\sum_{m\in f_{j}} p\left(m\right)g_{m}\left(\mu_{m},u\right) \\ \tilde{Q}_{f_{j}}\left(u\right)=\frac{1}{p\left(f_{j}\left|u\right.\right)}\sum_{m\in f_{j}} p\left(m\right)\left(\left(g_{m}\left(\mu_{m},u\right)-g_{f_{j}}\left(u\right)\right)\left(g_{m}\left(\mu_{m},u\right)-g_{f_{j}}\left(u\right)\right)^{T}+\tilde{Q}_{m}\left(u\right)\right) \end{matrix}$ A3.1

where the prior probabilities of model families are simply given by: $p\left(f_{j}\left|u\right.\right)=\sum_{m\in f_{j}} p\left(m\left|u\right.\right)$. The Laplace-Chernoff risk associated with the selection of model families then simply derives from replacing the prior predictive densities of models with those of model families, as given in Equation A8. We use examples of this in the main text.
